# A Review of Myositis-Associated Interstitial Lung Disease

**DOI:** 10.3390/jcm13144055

**Published:** 2024-07-11

**Authors:** Renuka Kannappan, Raagni Kumar, Kimberly Cichelli, Lawrence H. Brent

**Affiliations:** 1 Department of Internal Medicine, Temple University Hospital, Philadelphia, PA 19140, USA; 2 Section of Rheumatology, Temple University Hospital, Philadelphia, PA 19140, USA

**Keywords:** interstitial lung disease, idiopathic inflammatory myopathies, myositis, polymyositis, dermatomyositis, antisynthetase syndrome, MDA5

## Abstract

There is a well-established relationship between different subsets of idiopathic inflammatory myopathies (IIMs, myositis) and interstitial lung disease (ILD), with lung complications sometimes presenting prior to myopathic manifestations. The subtypes of myositis include those that are strongly associated with ILD, such as polymyositis (PM) and dermatomyositis (DM). Research has shown that in certain patients, these can then be further divided into subtypes using myositis-specific antibodies (MSAs), which are specific for myositis, and myositis-associated antibodies (MAAs), which can be found in myositis in overlap syndromes with other connective tissue diseases (CTDs). Notably, certain MSAs and MAAs are associated with ILD in patients with myositis. The clinical presentations of ILD in patients with myositis can vary widely and can be insidious in onset and difficult to diagnose. As ILD can progress rapidly in some cases, it is essential that clinicians are able to identify and diagnose ILD in patients with myositis. For this reason, the aim of this review is to highlight the clinical features, diagnostic criteria, important histopathologic, laboratory, and radiographic features, and treatment modalities for those patients with myositis-associated ILD.

## 1. Introduction

There is a well-established relationship between idiopathic inflammatory myopathies (IIMs, also referred to as myositis) and ILD, particularly with certain subtypes, with lung complications sometimes presenting prior to myopathic manifestations [1,2]. Myositis is a general term that includes many types of inflammatory diseases of the muscle. In this review, the term myositis will be used to include autoimmune myopathies. The subtypes of myositis include those that are strongly associated with ILD, such as polymyositis (PM) and dermatomyositis (DM), with recent review studies showing a prevalence of ILD in PM and DM that may be over 40% [3,4]. These can then be further divided into subtypes using myositis-specific antibodies (MSAs), which are specific for myositis and exclusive to a specific type, and myositis-associated antibodies (MAAs), which can be found in myositis in overlap syndromes with other connective tissue diseases (CTDs). Notably, there is a high prevalence of ILD associated with several MSAs, most strongly with anti-melanoma differentiate factor 5 (MDA5) and the anti-aminoacyl-tRNA synthetase antibodies (ASAb), which help to define antisynthetase syndrome (ASyS) [1]. The prevalence of ILD in MDA5+ patients varies widely across cohorts, ranging from 40 to 100% [5]. Among patients with ASyS, the prevalence of ILD ranges from 70 to 95% depending on the cohort [6]. For the purposes of this review, Bohan and Peter’s 1975 classification criteria for PM and DM, as well as the EULAR/ACR 2017 classification criteria for adult and juvenile myositis and their major subgroups, were utilized in describing the patient population of those affected by myositis-associated ILD, although it is worth noting that MSA and MAA are not included in either criteria [3,7,8]. Clinical presentations of ILD in patients with myositis can vary widely and can be insidious in onset and difficult to diagnose [8,9,10]. ILD can progress rapidly in some cases, so it is essential that clinicians are able to identify and diagnose ILD in patients with myositis. The aim of this review is to highlight the clinical features, diagnostic criteria, important histopathologic, laboratory, and radiographic findings, and treatment modalities for those patients with myositis-associated ILD. To better emphasize these points, we will feature three case reports of patients with myositis-associated ILD.

## 2. Clinical Cases

### 2.1. Case Report 1

**Figure 1:**
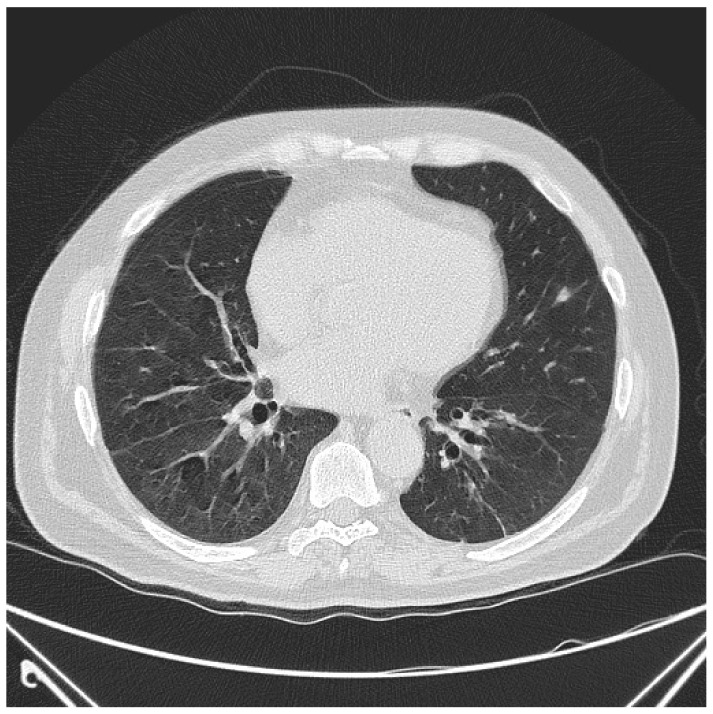
CT of the chest in the lower lung fields showing scattered ground glass opacities and peribronchial inflammation of the lower lung fields.

First, we present a case of ASyS with DM, anti-Jo-1+, and ILD. This 47-year-old female has a past medical history of hypertension, type 2 diabetes mellitus, chronic kidney disease, hyperlipidemia, obstructive sleep apnea, and fibromyalgia, and she was diagnosed with DM and ILD in 2010. Manifestations at the time of diagnosis included proximal muscle weakness, facial rash, shortness of breath, and polyarthritis. The physical exam was pertinent for a heliotrope rash, faint rash of mechanic’s hands, mild hip flexor weakness on the right side, and right elbow synovitis. Laboratory findings included elevated creatinine kinase (CK) of 1031 U/L (upper limit of normal [ULN] 143 U/L), aldolase 13.4 U/L (ULN 8.1 U/L), positive anti-Jo1, ANA at a titer of 1:80 with a speckled pattern, and positive anti-SSA. Imaging with a CT of the chest showed interstitial changes, scattered ground glass opacities, and peribronchial inflammation (Figure 1). Pulmonary function tests (PFTs) showed a restrictive pattern: FEV1 69%, FVC 74%, FEV1/FVC 78%, DLCO/VA 5.73 mL/mmHg/min/L. She had reported a prior abnormal electromyography (EMG) at another facility; however, those reports were not available. A repeat EMG at the time of diagnosis was normal. Transthoracic echocardiogram showed a normal ejection fraction of 50–55% and no evidence of pulmonary hypertension. She was started on prednisone 20 mg daily. Since her diagnosis, she has been treated with prednisone, azathioprine, and then rituximab, which she received in 2015 due to an inability to wean off steroids and was able to discontinue steroids. She is currently treated with mycophenolate mofetil with stable-disease lung disease and inactive myositis. 

### 2.2. Case Report 2

**Figure 2:**
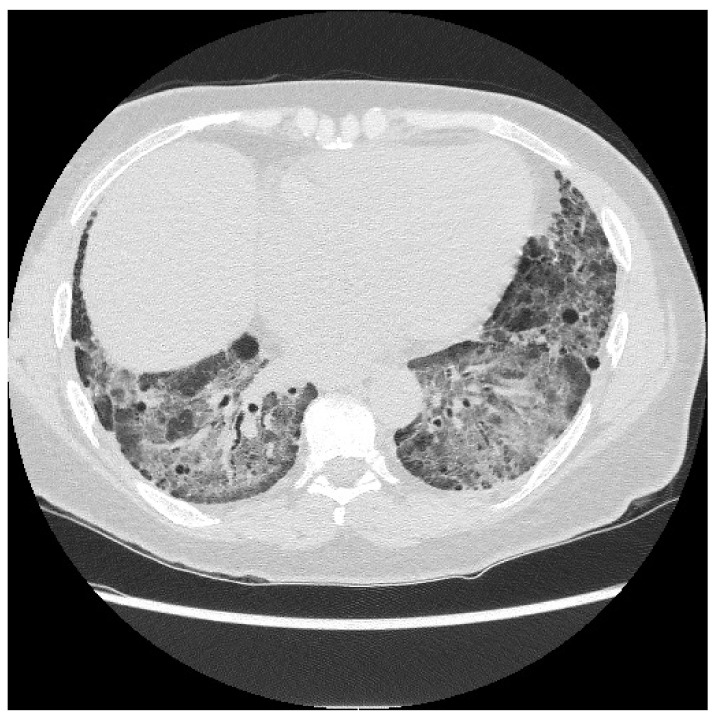
CT of the chest in the lower lung fields showing interstitial lung disease with fibrosis mostly in the lower lung fields.

Second, we present a 49-year-old female with ASyS with PM, anti-PL-12+, and ILD. Her past medical history was pertinent for hypertension, gastroesophageal reflux disease, hyperparathyroidism, end-stage renal disease due to hypertension, and renal transplant in August 2023. She was diagnosed with PM and found to have ASyS in 2015, with manifestations including myalgias with proximal muscle weakness of the upper and lower extremities, dyspnea on exertion and at rest, Raynaud’s symptoms, sicca symptoms, dysphagia, fatigue, weight loss, and alopecia. Laboratory studies showed an elevated CK of 949 U/L (ULN 230 U/L), positive ANA at a titer of 1:320 with a speckled pattern, positive rheumatoid factor (RF), anti-RNP, and anti-PL-12. Imaging with a CT of the chest showed fibrosis in the lower lung fields and interstitial changes in a nonspecific interstitial pneumonia (NSIP) pattern (Figure 2). PFTs showed a restrictive pattern, with FEV1 58%, FVC 51%, FEV1/FVC 92, and DLCO/VA 3.39 mL/mmHg/min/L. Transthoracic echocardiogram showed a normal ejection fraction of 70%, a small posterior pericardial effusion, and normal right ventricular systolic function, with no evidence of pulmonary hypertension. She was initially seen during a hospital admission and was started on high-dose corticosteroids with a taper after discharge. She was initially treated with azathioprine and nintedanib. She is currently on prednisone (now continued due to renal transplant), mycophenolic acid, hydroxychloroquine, tacrolimus (for renal transplant), and pirfenidone. At the time of writing this manuscript, she remains stable from both a pulmonary and renal standpoint. 

### 2.3. Case Report 3

**Figure 3:**
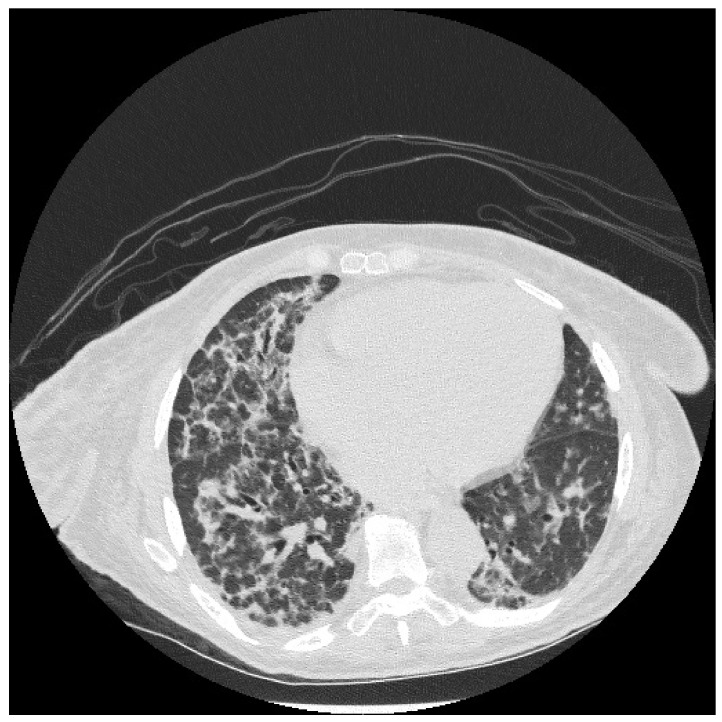
CT of the chest in the lower lung fields with organizing pneumonia with interstitial reticulations, ground glass opacities, peribronchial inflammation, and consolidation.

Lastly, we present a case of anti-MDA5-positive DM with ILD. The patient is a 68-year-old female with a past medical history of hypertension, hyperlipidemia, and chronic kidney disease (stage 3), who was diagnosed with DM in 2017, with manifestations of severe skin disease, muscle weakness/myositis, shortness of breath, dry cough, and weight loss. She was later found to have concomitant non-ischemic cardiomyopathy, which was felt to be due to her underlying DM. Physical exam showed a heliotrope rash, mechanic’s hands, and decreased muscle strength of both proximal upper and lower extremities. Serologies were pertinent for a mildly elevated CK at 280 U/L (ULN 140 U/L), positive anti-MDA5, positive ANA at a titer 1:640 with a speckled pattern, anti-SSA, and anti-Ro52. Imaging with CT of the chest showed interstitial changes with organizing pneumonia with interstitial reticulations, ground glass opacities, peribronchial inflammation, and consolidation more prominent in left lower lobe (Figure 3). PFTs were ordered but were never obtained. Transthoracic echocardiogram in 2019 showed evidence of heart failure with reduced EF (30–35%) and moderate diffuse hypokinesis, but no evidence of pulmonary hypertension; a follow-up transthoracic echo in 2020 showed improved EF to 45–50%. EMG testing of the upper extremities in 2017 revealed moderate median neuropathies across the wrists, with the right worse than the left. She was started on corticosteroid therapy and intravenous immunoglobulin (IVIg) for newly diagnosed DM. A malignancy workup was performed and was negative at the time of diagnosis. Since her diagnosis, she has been treated with intravenous cyclophosphamide and then mycophenolate mofetil. Unfortunately, the patient was lost to follow-up after April 2020, but at last visit she was taking mycophenolic acid, prednisone, and she was receiving monthly IVIg with plans to hold IVIg until the next follow-up appointment. At the last rheumatology and pulmonology visits in mid-2020, the patient was doing well, with stable muscle, skin, and lung disease. 

## 3. Myositis: Polymyositis and Dermatomyositis

### 3.1. Classification of Idiopathic Inflammatory Myopathies (Myositis)

The classification of myositis had undergone many changes since Bohan and Peter’s first attempt in 1975 [3]. See Table 1 for the initial classification schema. Over time, the addition of pathology and autoantibodies has added information that resulted in various updates to the classification of myositis over time; Table 2 refers to newer classification criteria that has emerged. Pathologic studies added two new types of myositis: immune-mediated necrotizing myositis (IMNM) and inclusion body myositis (IBM), which are generally not associated with ILD. Autoantibodies help define another syndrome: antisynthetase syndrome (ASyS), which is highly associated with ILD; Table 3 summarizes classification of myositis with various myositis-specific antibodies.

### 3.2. Epidemiology

PM and DM are autoimmune myopathies characterized by various degrees of muscle inflammation, and in DM, typical cutaneous lesions. Epidemiological studies of myositis tend to involve small population-based samples and use medical records and muscle biopsy to identify patients. Incidence rates for PM and DM range between 4.27 and 7.89 per 100,000 person-years and prevalence ranges from 9.54 to 32.74 cases per 100,000 individuals [13]. Moreover, van der Muelen et al. attempted to accurately characterize the prevalence of myositis. In a retrospective study, 76/165 patients were thought to have PM using diagnostic criteria; however, at the 4-month follow-up, only 4 of these patients were shown to have PM via histopathologic data [14]. This demonstrates that PM is rare and often misdiagnosed. PM and DM occur more frequently in women and people of Black ethnicity. DM affects both children and adults, whereas PM rarely occurs in the pediatric population [13].

### 3.3. Etiology and Pathogenesis

As in other systemic autoimmune diseases, myositis is associated with a variety of autoantibodies, being found in about 50% of such patients. These can be divided into two subsets: MSAs, which are specific for myositis, and MAAs, which are found in patients with myositis in overlap with other connective tissue diseases. Although these autoantibodies are not currently included in the diagnostic criteria, they are associated with different clinical phenotypes, as demonstrated in Table 4. Of note, several MSAs, notably the ASAb and MDA5+, are characterized by a high prevalence of ILD [1]. 

The pathogeneses of PM and DM have considerable overlap and share important features [11]. In PM and DM, skeletal muscle cells (myocytes) aberrantly express MHC class I receptors on their cell surface. In PM, CD8+ T cells (cytotoxic) are expanded and bind to these MHC class I molecules on the myocytes. The cytotoxic T cells possess perforin containing cytoplasmic granules, which when released induce necrosis of the muscle cells. CD4+ T cells (helper) activate B cells to produce autoantibodies. In DM, CD4+ T cells play a prominent role and after being activated, produce cytokines and activate B cells. Immunoglobulin and complement proteins are found in the vessels of muscle tissue from these patients, which may help to promote muscle damage.

Plasmacytoid dendritic cells (pDC) are also present in the muscle and skin of patients with DM and produce type I interferons, which promote inflammation and T and B cell activation [15,16]. Interferons (IFN), including IFN-α and IFN-β, activate inflammatory and immunologic pathways, promoting antibody production [11]. Activated B cells develop into plasma cells and produce autoantibodies. The exact role of the MSAs found in patients with PM and DM is not clear. ASAb may play a role in the pathogenesis of ASyS (see Section 4. Antisynthetase syndrome) [17].

Toll-like receptors (TLRs) are expressed on myocytes in patients with PM and DM. The expression of TLRs on myocytes was associated with increased tissue expression of INF-γ, IL-4, IL-17, and TNF-α [18]. This suggests that activation of myocytes through TLRs recruits immune cells, leading to the production of pro-inflammatory cytokines. Numerous studies have revealed similar cytokine and chemokine profiles in muscle tissue from patients with PM and DM, suggesting that activated CD4+ T cells may be involved in both disease processes. Other studied mechanisms of pathogenesis include the adaptive immune system; B cells activated to plasmablasts and plasma cells to produce autoantibodies and T cells to produce IL-6, IL-17, IFN-γ, and BAFF; and the innate immune system, including macrophages and pDC, producing IL-1, IL-6, IL-15., IL-23, and IFN-α and γ [19,20].

### 3.4. Diagnostic Criteria

Myositis includes a group of rare systemic autoimmune diseases characterized by muscle weakness and inflammation, as well as involvement in the skin, joints, lungs, heart, and other major organs. EULAR/ACR classifies myositis as DM, PM, inclusion body myositis (immune-mediated necrotizing myopathy), amyopathic DM, and juvenile DM [7].

Bohan and Peter were the first to develop commonly used criteria for PM and DM in 1975 [3]. There are five major criteria:

1. Symmetric proximal muscle weakness
2. Muscle biopsy evidence of necrosis and inflammation
3. Elevation of serum skeletal-muscle enzymes
4. Electromyographic myopathic changes
5. Pathognomonic rashes of DM

The first four criteria are deemed definitive for PM. The fifth criteria must be present in addition to at least three other criteria for a definitive diagnosis of DM. While used very frequently, these criteria do not define inclusion body myositis, amyopathic DM, and juvenile DM. The latter are further specified in the EULAR/ACR classification [7].

### 3.5. Clinical Features

For the purposes of this review, Bohan and Peter’s criteria are still very helpful for identifying PM and DM. In practice, both present with symmetric weakness of the proximal limb and girdle muscles. Patients may complain of difficulty getting up from a chair, climbing stairs, reaching their arms up, or getting out of a car. The weakness usually presents slowly over a few months and can relapse and remit [3,4,21,22]. If left untreated, the symptoms can be disabling.

DM is identified by its pathognomonic rashes: Gottron papules (erythematous patches over the extensor surfaces of the hand joints as well as knees, elbows, and ankles), heliotrope (lilac discoloration and erythematous periorbital edema), shawl sign (erythematous eruption over sun-exposed back), “V” sign (erythematous eruption on the upper chest, Holster sign (erythematous rash over the later hips and thighs), and mechanic’s hands (fissuring of palms and fingertips with irregular and thick cuticles) [3,22].

Laboratory findings in patients with PM and DM include skeletal muscle enzymes, which are often elevated, including creatine phosphokinase (CK), aldolase, aspartate aminotransferase (AST), alanine transaminase (ALT), and lactic dehydrogenase (LDH) [3,21,22]. More recently, the detection of myositis-specific autoantibodies (MSAs) and myositis-associated antibodies (MAAs) can aid in the diagnosis and correlate with the phenotype of disease [22,23]. As mentioned previously, MSAs and MAAs are not included in either Bohan and Peter’s criteria or the 2017 EULAR/ACR criteria, indicating that new criteria might be needed [3,7,8].

Diagnostic tests are not always necessary in making the diagnosis of PM or DM but can assist if the diagnosis is unclear. Electromyographic (EMG) changes consist of polyphasic short low amplitude units, spontaneous fibrillations, and positive sharp waves [8,21]. Muscle histopathology can differentiate between PM and DM. PM is characterized by endomysial infiltration throughout the muscle fascicle, which surround and invade muscle fibers. DM is characterized by perimysial infiltration around the fascicles and also perivascular inflammation. This grouping facilitates microinfarcts within the muscle, leading to atrophy and fibrosis [9,21]. The inflammatory infiltrate in PM consists of CD8+ T cells and macrophages, while in DM there are CD4+ T cells, B cells, macrophages, and plasmacytoid dendritic cells and there is vascular deposition of immunoglobulin and complement proteins [19,24].

In PM and DM, patients may also present with extra-muscular manifestations such as arthritis, Raynaud’s phenomenon, esophageal dysfunction, cardiac dysfunction, pulmonary disease, and vascular disease in the form of pulmonary arterial hypertension [3,4,21,22]. The prevalence of malignancy is increased in patients with PM and DM, more so with DM; therefore, age-appropriate cancer screening is recommended [9,25].

## 4. Antisynthetase Syndrome

### 4.1. Epidemiology

Antisynthetase syndrome (ASyS) was first described by Marguerite and coworkers in 1990 [26]. It is classified as a chronic autoimmune disease that falls under the umbrella term of myositis, which also includes PM, DM, and inclusion body myositis. ASyS is defined by the presence of ASAb and clinical features including fever, myositis, ILD, mechanic’s hands, Raynaud’s phenomenon, and inflammatory arthritis [27]. ASyS patients may also have pulmonary arterial hypertension [6]. Patients with ASyS may present with a variety of clinical manifestations, including myositis (56%), inflammatory arthritis (64%) that may be misdiagnosed as rheumatoid arthritis, ILD (51%), fever (26%), mechanic’s hands (20%), Raynaud’s phenomenon (24%), and the complete triad of arthritis, myositis, and ILD (20%) [28]. In patients with established disease, 79% of patients had myositis, 84% had ILD, and 60% had the complete triad.

There are several challenges with the diagnosis of ASyS. First, the disease process can have varied presentations and clinical manifestations of differing severity. Second, cohort studies have indicated that 25–35% of patients diagnosed with PM or DM have antisynthetase antibodies (ASAbs) [6]. Furthermore, it is likely that many patients with ASyS remain undiagnosed, especially those who present with arthritis or ILD without myositis [26]. The burden is placed on the physicians to whom these patients present to be familiar with the variety of clinical presentations to include ASyS in their differential diagnosis, especially for ILD of unknown origin. As a syndrome, ASyS is poorly defined, but what we do know is that women are more likely to be affected than men (greater than 2:1), with the age of onset among adults varying from 19 to 82, with a mean age at onset ranging from 43 to 60 years, and patients of Black ethnicity are thought to have increased severity of ILD [29]. 

### 4.2. Etiology and Pathogenesis

ASyS is established by the presence of autoantibodies against cytoplasmic enzymes specialized for the attachment of an amino acid to its tRNA molecule, also known as an aminoacyl-tRNA synthetase. As there are 20 essential amino acids present in humans, there are, in theory, 20 ASAbs. However, there are only eight identified ASAb at this time. Anti-Jo1 is the most common of these and is found in approximately 20–25% of patients with myositis [30]. Table 5 lists the eight known ASAbs as well as their antigens, prevalence in myositis as a whole and in ASyS specifically, prevalence of associated clinical features as well as other commonly seen manifestations listed in order of the most to least commonly observed [6,27,31,32].

It has been hypothesized that viral pathogens in the lung induce the activation of cytotoxic T cells, natural killer (NK) cells, and neutrophils. These cells produce granzyme B, which effects the cleavage of histadyl-tRNA synthetase. The resulting peptides are then taken up by antigen-presenting cells (APC) in local lymph nodes. APC present antigens to T helper cells and B cells, activating them and resulting in the production of autoantibodies, ASAb [17,30]. Immunization of mice with histadyl-tRNA synthetase (Jo-1) induced inflammation in the muscles and lungs associated with the production of anti-Jo-1 antibodies [33]. The inflammation is mediated by the innate and adaptive immune responses [34]. Histadyl- and asparaginyl-tRNA synthetase (Jo-1 and KS, respectively) have been shown to stimulate T cells and dendritic cell, promoting immune mediated inflammation [35].

Anti-Jo-1 is strongly associated with HLA-B*08:01 and HLA-DRB1 03:01 [30]. In organs, such as the lungs or muscles, with minor tissue injury resulting in autoantigen expression (peptides from aminoacyl-tRNA synthetase), an immune-mediated inflammatory reaction may occur, resulting in tissue damage. pDC are activated, producing IFN-α, which is immunostimulatory and may contribute to MHC class I expression on the surface of myocytes [17]. Other potential pathogenic mechanisms involve reduced NK cell function, resulting in impaired IFN-γ production and NETosis [30].

Regarding the immunologic process of ASyS-associated ILD, Zhou et al. shed some light. They demonstrated that naturally expressed splice variants of histidyl-tRNA synthetase are robust targets for anti-Jo-1 antibodies. They noted that these splice variants are expressed endogenously at low levels and that there is increased expression in lung tissue as compared with tissue from other organs, which can explain the pathogenesis of pulmonary disease, although further work is needed to better understand the specific role that antisynthetase enzymes play [36].

ASyS-associated ILD is likely mediated by the innate immune response and associated with pro-inflammatory cytokines. Gono et al. evaluated the cytokine profiles of 78 patients with myositis-associated ILD; they found that the serum levels of the pro-inflammatory cytokines IL-6, IL-8, TNF-α, and IFN-γ-induced protein 10 were significantly elevated in patients with ILD compared to controls [37].

### 4.3. Diagnostic Criteria

In the first Bohan and Peter classification criteria for myositis, as well as the 2017 ACR/EULAR classification criteria, ASyS is generally categorized by DM or PM. However, there are new suggestions that although ASyS shares features with DM and PM, it has its own unique features, which suggest that is a separate disease entity within myositis. There are two major proposed classification criteria for ASyS, both of which require the presence of ASAb as well as specific clinical features [38]. These are described in Table 6.

### 4.4. Clinical Features

The classic clinical triad of ASyS includes myositis, arthritis, and ILD; this accounts for up to 90% of cases. Other cardinal clinical features include fever, mechanic’s hands, Raynaud’s phenomenon, and pulmonary arterial hypertension (PAH) [6,30]. Looking at the timeline and chronological order of the clinical presentation of ASyS, Baccaro et al. found that muscle disease, arthritis, and fever predominated as the initial symptoms, whereas mechanic’s hands, Raynaud’s phenomenon, and pulmonary manifestations appeared later [41]. ASyS-associated ILD will be discussed in further detail below as it is the focus of this paper; however, we will now briefly discuss the other commonly seen clinical manifestations. ASyS patients may present with ILD or arthritis without myositis and may never be diagnosed because autoantibody testing may not include ASAb. The prevalence of patients such as this is unknown.

Muscle disease is seen in more than 90% of patients with ASyS, more specifically associated with anti-Jo-1 antibodies. It is infrequently subclinical with a transient CK elevation as the only presenting abnormality. However, much more commonly, patients develop profound muscle weakness and pain. Weakness of the upper esophagus and pharynx may cause dysphagia and make these patients more susceptible to aspiration pneumonia. This may also cause dysphonia.

**Figure 4:**
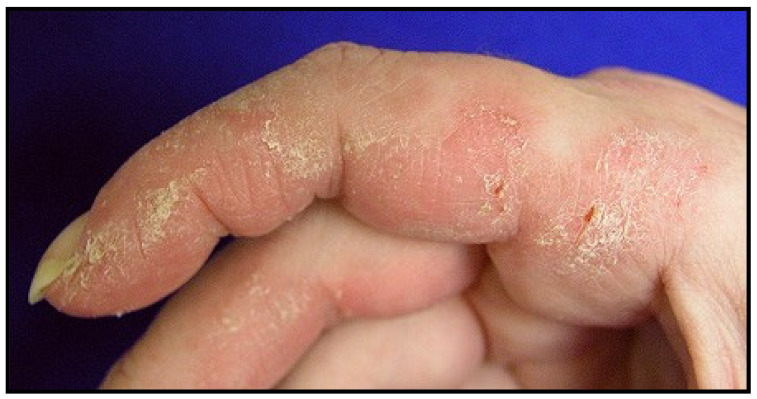
Mechanic’s hands, courtesy of C.V. Oddis.

Arthritis and arthralgias are common and seen in approximately 50% of patients. The most common form is a symmetric non-erosive polyarthritis of the small joints of the hands and feet. Mechanic’s hands are seen in approximately 30% of patients. This is a dermatologic phenomenon where the skin of the tips and margins of the fingers, predominantly on the radial side of the index fingers, becomes thickened, hyperkeratotic, and fissured (see Figure 4). Biopsy of the skin will demonstrate an interface psoriasiform dermatitis. Raynaud’s phenomenon develops in about 40% of patients. Most commonly, nailfold capillary abnormalities will be seen, but severe digital ischemia is extremely uncommon. PAH develops in approximately 25% of patients; however, this is likely an underestimation as several patients do not undergo right heart catheterization, which would determine the severity of their PAH [27].

## 5. Anti-MDA5 Amyopathic DM

### 5.1. Epidemiology

MDA5+ DM is a rare but distinct subtype of myositis. As discussed in previous sections, the discovery of MSAs allowed for better classification of the clinical phenotypes of patients presenting with myositis. One of these MSA, the MDA5 antibody, was originally discovered in 2005 [5]. Before this, also known as the pre-MDA5 phase, the nomenclature was clinically amyopathic DM (CADM). Since 2005, the terminology has changed to MDA5+ DM. Although there is a significant overlap with CADM and MDA5+ DM, this disease process differentiates itself by its increased risk of rapidly progressive interstitial lung disease (RP-ILD). The classic presentation of MDA5+ DM also differs from DM, with the predominance of pulmonary, skin, articular, or vascular symptoms [42]. The prevalence of MDA5+ DM varies based on the region, with the prevalence in European studies ranging from 1.3 to 10%, while in Asian cohorts it is higher at 15–36.6% of patients with myositis [43,44,45]. Similar to other autoimmune diseases, MDA5+ DM occurs mainly in women, with a female/male ratio of ~2:1 [42]. The prevalence of ILD in MDA5+ DM ranges from 50 to 100%, with the variation likely reflecting population discrepancies [46]. RP-ILD, which has no exact definition but is intuitively thought of as measurable progression within a short period of time since the onset of ILD, has a prevalence of 38–71% [46].

### 5.2. Etiology and Pathogenesis

The etiology of MDA5+ DM, as with all the other types of myositis, is largely unknown; however, there are several studies demonstrating that interactions between environmental and genetic factors are implicated in the disease process. Multiple immune cells, including B cells, T cells, neutrophils, macrophages, and NK cells, have been shown to be involved in the pathogenesis of MDA5+ DM [5].

Environmental risk factors include the picornaviruses (hepatitis A, coxsackie B, enterovirus, rhinovirus); the mechanism involves the *IFIH1* gene, which encodes MDA5 and serves as the key protein sensor of viral double stranded DNA. These viruses can activate MDA5, with the production of type 1 interferons (IFNα and IFNβ) as well as other inflammatory cytokines involved in the antiviral response [46]. A study performed by Nishina et al. in 2020 further demonstrated the environmental influence by discovering that MDA5+ DM-ILD occurred predominantly in the winter months of October–March in individuals who resided near freshwater [47].

There are several genetic risk factors as well as immune cell dysregulation that play a role in its pathogenesis. In Chinese patients, HLA-DRB1*0401 (odds ratio 16.2) and HLA-DRB1*1202 (odds ratio 3.1) are associated with an increased risk of MDA5+ DM [48] and HLA-DRB1*0901 is associated with a worse prognosis [49]. In Japanese patients, HLA-DRB1*0101 and *0405 are associated with an increased risk of MDA5+ DM patients [50].

The skin involvement of MDA5+ DM is thought to be related to activation and infiltration of pDC and overexpression of type 1 IFN. Keratinocytes are also a source of type I IFNs. Type I IFN activates endothelial cells and macrophages to produce CXCL10 (IP-10). CXCL10 is a chemoattractant for T cells, macrophages, NK cells, and dendritic cells. CD8+ cytotoxic T cells attack keratinocytes [5,42].

With regards to RP-ILD, MDA5 expression on epithelial cells in the presence of autoantibodies may trigger the inflammatory reaction. Type I IFN activates endothelial cells to produce CX3CL1. CX3XL1 is a chemoattractant for T cells and monocytes. CD4+ T cells producing various cytokines recruit CD8+ cytotoxic T cells. The pro-inflammatory proteins and cytokines produced include ferritin, TGF-β, and IL-13, which activate alveolar macrophages and interstitial fibroblasts, resulting in fibrosis [5,42]. The presence of anti-MDA5 along with elevated CRP and KL-6 levels predicts a worse prognosis for ILD in patients with IIM [51].

### 5.3. Diagnostic Criteria

MDA5+ DM, although it is a unique disease process, is still classified as a subtype of DM. There are no universally recognized classification criteria for MDA5+ DM. When looking at the previously described 2017 EULAR-ACR classification criteria for myositis, DM can be classified into typical DM and amyopathic DM [7]. Accurate detection of anti-MDA5 antibodies is critical for diagnosis. As mentioned previously, there are no current criteria that utilize MSAs as part of the diagnosis; however, in 2018, the European Neuromuscular Centre grouped DM by MSAs, including MDA5+ DM characterized by Gottron’s and heliotrope signs. However, some patients do not present with the aforementioned DM skin manifestations and will instead present with pulmonary involvement. These patients are often diagnosed with MDA5+ DM in clinical practice; however, they technically do not meet the existing classification criteria [5]. This emphasizes the importance of an update to the criteria to keep up with the latest discoveries in these disease processes.

### 5.4. Clinical Features

MDA5+ DM has a heterogenous clinical phenotype affecting various organs in the body, primarily the skin, mucosa, joints, and lungs, and less so the muscle [52]. ILD is the most serious manifestation of MDA5+ DM and will be discussed in a later section, so we will now briefly discuss other commonly seen manifestations.

**Figure 5:**
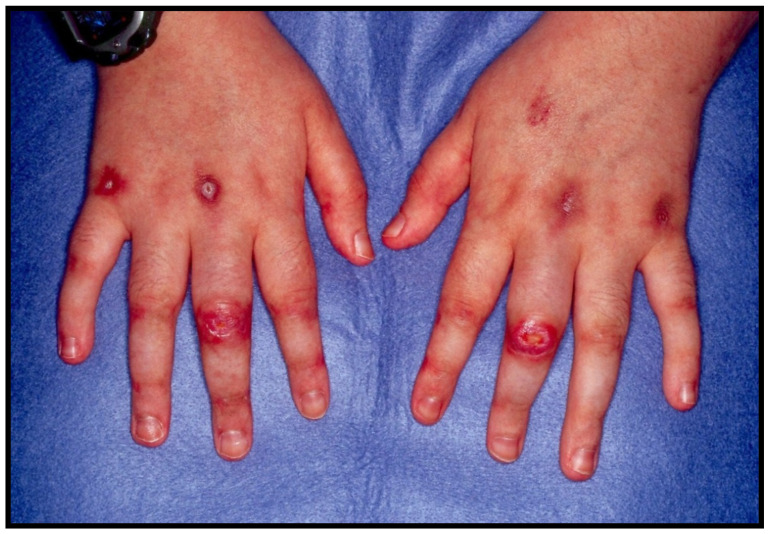
Cutaneous ulceration overlying Gottron papule in MDA5+ DM.

The dermatological findings can be divided into classical and pathognomonic. Classical manifestations on the skin appear in 60–70% of patients with MDA5+ DM and include Gottron’s papules and heliotrope rash; these may look familiar as they are the cutaneous symptoms included in the 2017 EULAR/ACR classification criteria for myositis discussed earlier [53]. Pathognomonic findings revolve around mucocutaneous findings, which are more specific for MDA5+ DM when compared to DM alone [5]. Skin ulceration is the most common of these mucocutaneous findings, occurring in 82% of cases. They often present at specific sites such as an overlying Gottron sign on the proximal interphalangeal (PIP) or metacarpophalangeal (MCP) joints of the fingers, elbows, shoulders, knees, ankles, digital pulp and nail folds; refer to Figure 5 [52]. They are usually deep, punched out, and painful. This manifestation is considered the strongest predictor of ILD in patients with MDA5-DM [52]. Other skin findings include palmar papules, located on MCP and PIP joints, non-scarring alopecia, and calcinosis with fingertip ulcers [5].

Constitutional symptoms such as fever, fatigue, and unintentional weight loss are commonly present. Arthritis and arthralgias are seen in over 50% of patients with bilateral, symmetrical involvement of the small joints [53]. There are several cardiac manifestations reported, such as arrhythmias, conduction defects, pericarditis, and cardiomyopathies. Of note, muscle involvement, if present, is very mild, which can be confusing as myositis is present in the name of the disease; this is a key differentiating factor [5].

## 6. Interstitial Lung Disease in Myositis

ILD is the extra-muscular manifestation of interest in this review. It is a hallmark feature of all the types of myositis discussed in this review. ILD is a major cause of morbidity and mortality in patients with myositis [4,31,54,55,56]. In a large cohort of patients with myositis in Spain, ILD was associated with increased mortality, with a hazard ratio of 1.61 [56]. Recent reviews suggest that the global prevalence of ILD in individuals with PM and DM may be over 40% [3,4]. The prevalence of ILD ranges from 70 to 95% among patients with ASyS depending on the cohort [6]. The prevalence of ILD is anywhere from 40 to 100% in MDA5+ DM patient patients; the reported incidence of RP-ILD in MDA5+ DM varies widely across different cohorts and is notably higher in Asian populations when compared to European populations [5]. A study by Jin et al. demonstrated that 54% of patients with MDA5+ DM in China had RP-ILD [57]. With regards to MSAs and MAAs, there is heterogeneity in the prevalence and severity of ILD. Patients with anti-PL-7 and anti-PL-12 autoantibodies have a higher prevalence of ILD when compared to patients with anti-Jo-1. Furthermore, patients who do not have anti-Jo-1, particularly those with anti-PL-7 and anti-Ro52 antibodies, have been shown to have early, severe, and more rapidly progressive ILD [6]. Beyond antibodies, the main predictive factors for progressive pulmonary disease were male sex, age ≥ 55, low DLCO at diagnosis, decreasing FVC over time, presence of anti-Ro52 antibodies, muscle weakness, and higher fibrosis score on high-resolution computed tomography (HRCT) [29]. The Black race was found to be an independent risk factor for severe ILD in ASyS when compared to the white race; Black patients have been shown to have lower FVC and DLCO [58]. Patients with anti-MDA5 are more likely to have RP-ILD with an OP pattern than other myositis patients, especially in Asians [32]. Anti-Ro52 is an MAA that is associated with an increased risk of ILD in patients with myositis [59]. In patients with ASyS, the presence of anti-Ro52 was associated with an increased risk of ILD [60]. In patients with anti-MDA5, anti-Ro52 was associated with an increased risk of ILD and more severe disease [61]. Anti-Ro52 was associated with a higher frequency of RP-ILD in patients with anit-Jo-1 and anti-MDA5 [42,62]. It is imperative that clinicians can identify and diagnose ILD in patients with these inflammatory myopathies. Table 7 shows the frequency of various autoantibodies and their association with ILD.

### 6.1. Clinical Presentation in Myositis-Associated ILD

The clinical presentation of ILD in patients with myositis is very variable, often correlating with the autoantibody profile of the patient. Most often, patients have insidious pulmonary complaints, including dyspnea, exercise intolerance, and chronic dry cough; however, some may be asymptomatic [8,9,10]. Fine inspiratory crackles in the lung bases often described as dry crackles may be heard on auscultation and changes may be seen on plain chest radiographs; however, these are relatively insensitive findings and should not be used as screening tests [10,27]. Given the higher mortality and prevalence of ILD in patients with myositis, the recommended testing includes pulmonary function testing (PFTs) with DLCO as well as a 6 min walk test [8,63]. A restrictive lung pattern with low FVC, TLC, and DLCO is suggestive of ILD [63]. Following PFTs serially can be helpful in following ILD progression. The diagnosis of ILD is confirmed with HRCT of the chest, which is the gold standard [63]. Generally, any change in respiratory symptoms or decrease in FVC or DLCO should prompt imaging and further workup [8,9]. 

### 6.2. Histopathologic and Radiographic Findings in Myositis-Associated ILD

**Figure 6:**
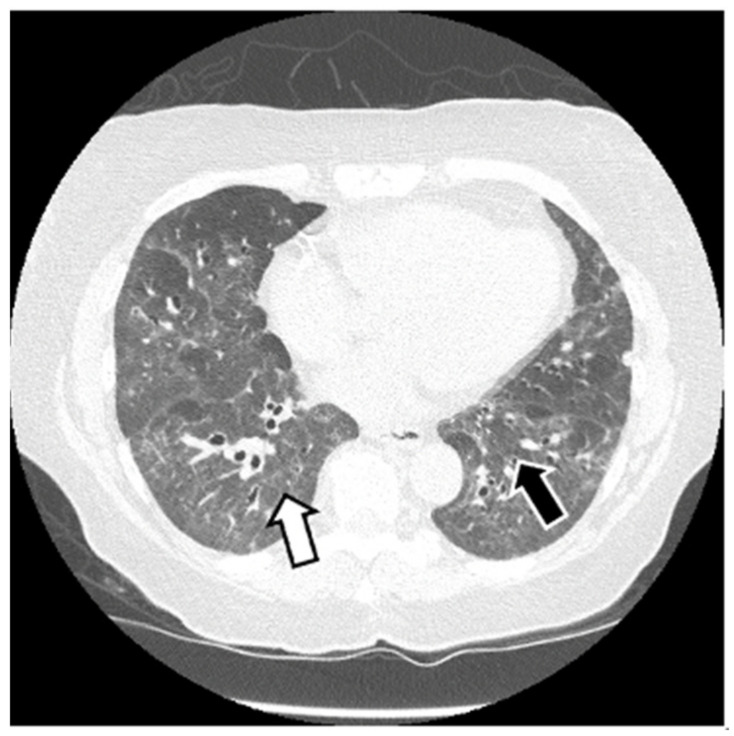
NSIP: diffuse GGOs in the lower lobes (white arrow) and traction bronchiectasis (black arrow) in a patient with ASyS with anti-PL-12 and anti-Ro52.

**Figure 7:**
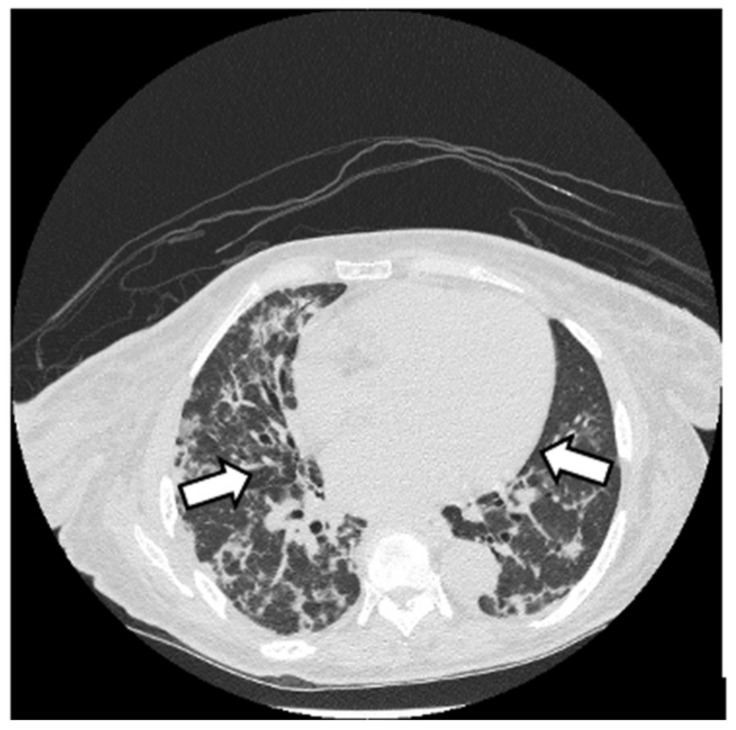
OP pattern with patchy consolidations (white arrow) in a patient with DM with anti-MDA5 and anti-Ro52.

**Figure 8:**
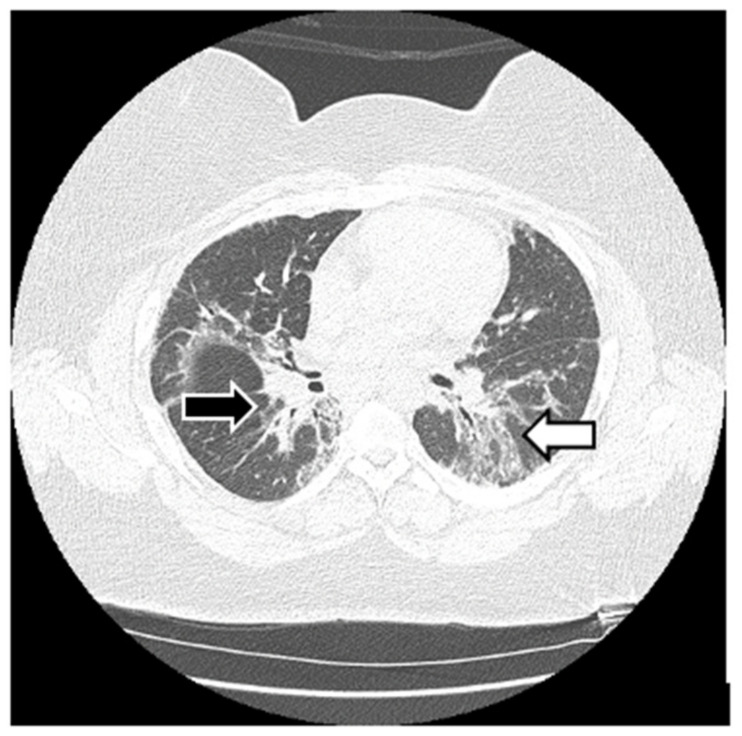
Mixed NSIP/OP pattern: GGOs peripherally (white arrow) and parenchymal bands (black arrows) in a patient with ASyS with anti-PL-12 and anti-Ro52.

**Figure 9:**
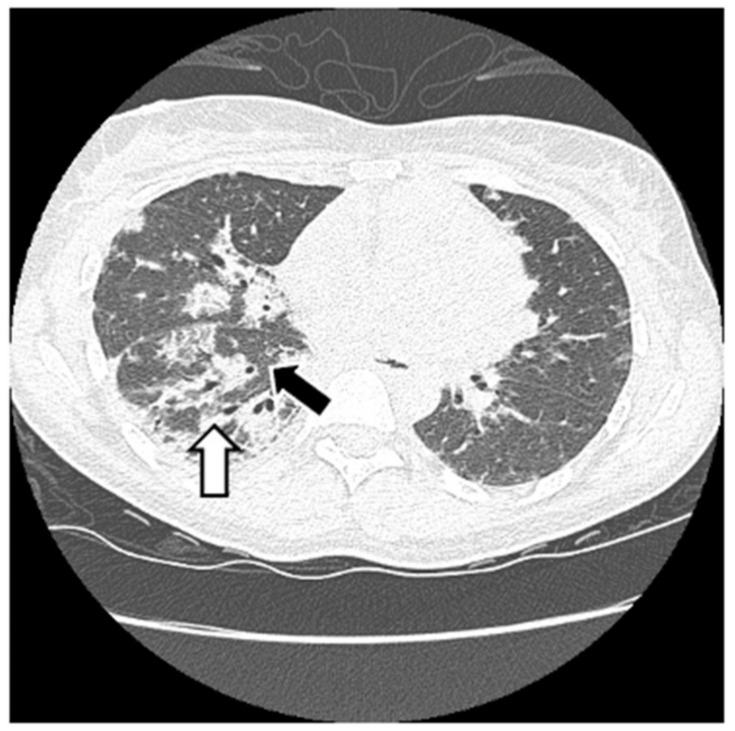
Fibrotic NSIP: fibrotic changes in the lower lungs (white arrow) and traction bronchiectasis (black arrow) in a patient with ASyS and anti-PL-7.

**Figure 10:**
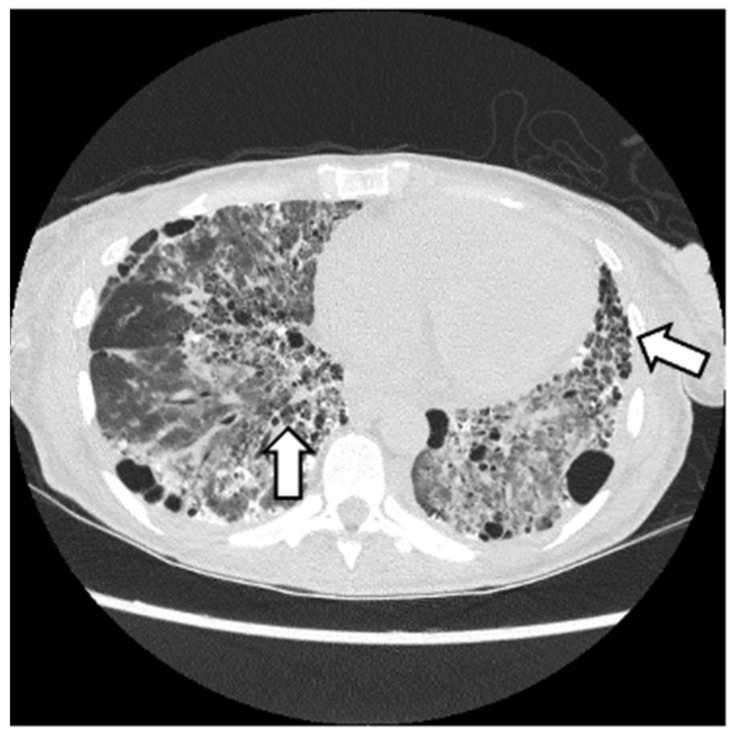
UIP: fibrotic changes with honeycombing in the lower lungs (white arrow) in a patient with ASyS and anti-Jo-1.

HRCT is used to confirm the diagnosis of ILD. The most common abnormalities seen in myositis-associated ILD on HRCT are cellular nonspecific interstitial pneumonia (NSIP), organizing pneumonia (OP), mixed NSIP/OP pattern, acute interstitial pneumonia (AIP), usual interstitial pneumonia (UIP), and fibrotic pattern. NSIP is characterized by bilateral, symmetrical ground glass opacities (GGOs) with a medial and basal distribution (Figure 6) [10]. The late stages of NSIP will have fibrotic changes and are referred to as fibrotic NSIP. OP often presents with air-space consolidations in a typically subpleural and peribronchial distribution (Figure 7) [10]. Mixed NSIP/OP occurs when consolidations are superimposed on GGOs (Figure 8) [10]. AIP is defined as bilateral patchy GGOs with associated areas of consolidation, but most importantly, symptom presentation within one month. Fibrotic NSIP has a pattern of lung involvement similar to NSIP but has a fibrotic appearance on HRCT (Figure 9) [10]. UIP is included in the fibrotic pattern of the disease as there is often honeycombing, traction bronchiectasis, irregular septal thickening and reticulations especially in the subpleural regions (Figure 10) [10,64]. Overall, the most common HRCT pattern is nonspecific interstitial pneumonia (NSIP); however, there have been studies that show that different MSAs and ASAbs are associated with different patterns of lung involvement [8]. In a report from a cohort study, patients with ASyS (anti-Jo-1, anti-PL-7, anti-PL-12, anti-EJ, anti-OJ) and ILD in China demonstrated that GGOs were very common being seen in >90% of patients, while reticulations were common occurring in 60–90% of patients. Honeycombing was uncommon with anti-Jo-1 (3%), more common with anti-PL-7, anti-PL-12, and anti-EJ (11–23%), and most common with anti-OJ (60%) [65]. NSIP was more common with anti-Jo-1, anti-PL7 and anti-EJ, while OP was common with anti-PL-12, and UIP was seen more often in anti-OJ patients [65].

The clinical manifestations and imaging findings of ILD, although quite similar among all the subtypes of IIM, do have their different clinical correlations. Tanizawa et al. demonstrated that lower consolidation and GGO patterns conferred higher short-term mortality in patients with PM/DM. AIP is most common in amyopathic DM with anti-MDA5 antibodies, less so in PM, and it is related to the development of diffuse alveolar damage [66]. Bonneffoy et al. discovered that when honeycombing is present on the initial CT scans of patients with DM, progressive pulmonary deterioration is more likely to occur [67]; these patients would benefit from more frequent follow-up to assess disease progression. Waseda et al. looked at 64 patients with ASyS-associated ILD. The most common HRCT features were GGOs in 98%, reticulations (67%), and consolidation (48%). The distribution is most commonly seen in lower lung fields (98.4%), around the bronchovascular bundles (73.4%), in the periphery (95.3%), and accompanied by a loss of volume in the lower lobes (89.1%) [45]. Hozumi et al. studied MDA5+ DM patients and found that the most common HRCT finding is what they labeled as unclassifiable. Imaging consisted of consolidations and GGOs associated with reticulations; per our definition, this would fall under the category of mixed NSIP/OP [68]. In other studies of MDA5+ DM, the most common ILD pattern was found to be OP (up to 50%), followed by mixed NSIP/OP (30%) and NSIP (20%) [69].

BAL and surgical lung biopsy are generally not indicated to diagnose myositis-associated ILD, largely due to concerns that such procedures could incite a disease flare. However, when a lung biopsy is obtained, the histopathology tends to correlate with HRCT findings. NSIP is the most common pathology observed in IMMs with a prevalence of 41% in one review; other pathologies include UIP (24%), organizing pneumonia (19%), and diffuse alveolar hemorrhage (14.5%) [2].

### 6.3. Treatment of Myositis-Associated ILD

There is no consensus-based guideline for the treatment of myositis-associated ILD; however, first-line treatment is universally considered to be glucocorticoids (GCs), followed by several steroid-sparing immunosuppressive agents. There are also no prospective, randomized studies to date that compare the efficacy of various treatment agents [70]. The most commonly used agents with their respective outcomes are listed in Table 8. This demonstrates an area where current myositis-associated ILD research is lacking; consequently, patients are often treated based on the physician’s clinical acumen and patients will usually require several treatment agents to prevent disease progression.

#### 6.3.1. Glucocorticoids

GCs are first-line therapy for myositis-associated ILD as providers are familiar with the medication, there is historical precedent, and the medications have a relatively fast onset of action. Doses range from 0.75 to 1.0 mg/kg/day [70]. Prolonged exposure to GCs is associated with significant side effects, including but not limited to weight gain, osteopenia/osteoporosis, hyperglycemia, and psychiatric problems. In addition, the response rate of myositis-associated ILD to GCs as monotherapy ranges from 37.5% to 52% [71]. Therefore, GCs are usually used in combination with additional agents early in the disease presentation.

#### 6.3.2. Mycophenolate Mofetil

Mycophenolate mofetil (MMF) is a first-line steroid-sparing agent used in the treatment of myositis-associated ILD. This is an immunosuppressive agent used in several rheumatological conditions, including systemic lupus erythematosus (SLE), IIM, and vasculitis. Fischer et al. found that after initiation of MMF, patients with myositis-associated ILD maintained stable lung function despite a decrease in the prednisone dose, which established MMF as an effective steroid-sparing agent [76]. Other small case series have also demonstrated that MMF is effective at improving lung function independent of prednisone [72]. The minimum efficacious dose is 2 g daily, and it can be up titrated to a maximum of 3 g daily [70]. Gastrointestinal side effects are common; less common side effects include cytopenias in the setting of bone marrow suppression and an increased risk of dermatologic malignancy; patients would benefit from regular monitoring of the CBC and LFT as well as yearly skin exams to look for suspicious lesions [70].

#### 6.3.3. Azathioprine

Azathioprine (AZA) is a commonly used steroid-sparing agent reported in the treatment of myositis-associated ILD. Marie et al. found that over half the patients receiving AZA as treatment for myositis-associated ILD with anti-Jo-1 antibodies demonstrated a positive clinical response [77]. Another retrospective study by Sharma et al. in 2017 with similar methods found a similar response rate of approximately 54% [72]. Huapaya et al. performed a retrospective analysis of myositis-associated ILD patients, of whom 66 received AZA monotherapy and 44 received MMF monotherapy. Although both groups demonstrated an improvement in the % predicted FVC, only the AZA group experienced an improvement in the % predicted DLCO [78]. Dosing is 1.5–2.5 mg/kg/day. The common adverse effects of AZA are similar to those of MMF, as described above: gastrointestinal toxicity, cytopenias, and elevated liver function tests [70].

#### 6.3.4. Calcineurin Inhibitors

Calcineurin inhibitors (CNIs) such as cyclosporine and tacrolimus work via T cell inhibition and are second-line steroid-sparing agents commonly used when treatment is refractory to MMF of AZA. Cavagna et al. performed a retrospective analysis of 17 patients with steroid-refractory anti-Jo-1 positive ILD and found that treatment with cyclosporine was associated with an average improvement in HRCT and PFTs at the 96-month follow-up [79]. Sharma et al. performed a retrospective analysis in 2017 of patients with myositis-associated ILD who received GCs along with either AZA, MMF, or methotrexate. Among the patients who failed to respond to this initial therapy, 94% demonstrated improvement in pulmonary function and a decrease in the prednisone dose by over 50% following the addition of tacrolimus [72]. For tacrolimus dosing, we recommend starting at 0.5–1 mg twice daily and adjusting to a serum trough of 5–10 ng/mL [70]. The most common adverse effect is renal dysfunction; therefore, it is imperative that the tacrolimus level is checked each week for the first four weeks, then checked every four weeks for close monitoring [70].

#### 6.3.5. Rituximab

Rituximab (RTX) is a chimeric monoclonal antibody that targets the CD20 antigen on B lymphocytes, resulting in peripheral blood B cell depletion. The benefits of RTX in myositis-associated ILD have been studied as an adjunctive therapy to the first-line agents described above. As discussed previously, the presence of Ro52 antibodies is associated with more severe ILD in patients with myositis-associated ILD. Improvement in ILD’s clinical symptoms was seen in all the patients in a study conducted by Bauhammer et al., who were anti-Jo-1+ and had high titers of Ro52 after completing treatment with RTX [80]. Another retrospective study by Allenbach et al. reported a 10% improvement in FVC in 50% of patients and stabilization in the remaining 40% of myositis-associated ILD patients who had initially failed first-line treatment and then subsequently completed RTX treatment [73]. A study by Andersson looked at RTX in a group of 24 patients with IIM who had ASyS-associated ILD refractory to first-line agents. After treatment with RTX, the group experienced a clinically significant improvement in the FVC, DLCO, and HRCT abnormalities [81]. Of note, the use of immunosuppressive agents prior to RTX treatment could have possibly confounded these results. Dosing is 1000 mg IV on day 0, then 1000 mg IV on day 14, which is repeated every 6 months [70]. Adverse events include a transfusion reaction, an increased risk of infection, hypogammaglobulinemia, and hepatitis B reactivation [70].

#### 6.3.6. Intravenous Immunoglobulin

Intravenous immunoglobulin (IVIG) has been used to treat inflammatory myositis, with improved muscle and dermatologic findings, and it is FDA-approved for the treatment of DM [82]. However, there is limited research on its efficacy in myositis-associated ILD. It is therefore listed as an adjunctive treatment option. The largest study to date was by Huapaya et al., who conducted a retrospective analysis looking at 17 patients with myositis-associated ILD, of whom 82% had refractory disease [83]. The disease was refractory to GCs and two alternate immunosuppressive agents. With the addition of IVIG, roughly 40% of patients experienced an increase in FVC by 10% and the mean dose of GCs decreased by more than 50% [83]. Dosing is 2 g/kg given over 2–5 days every 4 weeks [70]. Overall, IVIG is often added as salvage therapy.

#### 6.3.7. Cyclophosphamide

Cyclophosphamide (CYC) is an agent used to treat severe manifestations of various rheumatic conditions and is a third-line agent for the treatment of myositis-associated ILD. CYC has been used to treat scleroderma-associated ILD [84]. Evidence of the efficacy for CYC comes from a study by Yamasaki et al., which included 17 patients with myositis-associated ILD, 9 of whom had refractory, progressive disease [85]. When treated with CYC, six out of the seven patients requiring oxygen were able to be weaned off and half of the patients had an improvement in their vital lung capacity as well as HRCT scores after 6 months [85]. Moreno-Torres et al. performed an observational comparative study of 47 patients with myositis-associated ILD, evaluating the effect of the addition IV CYC to background therapy consisting of GC and immunosuppressive agents [75]. Over 50% of the patients had ASAb. The patients were followed for 12 months. The dose of CYC was 500 mg IV every 2 weeks for 6 doses (this could be extended for an inadequate response). Using the improvement of FVC > 10% as the primary outcome, 64% of the patients treated with CYC responded compared to 32% in the non-CYC group (*p* = 0.03). The patients with ASAb showed a better response than those without these antibodies. The CYC group also had a lower 6-month average dose of prednisone [75].

Of note, MDA5+ DM, which is associated with RP-ILD as we have discussed, does not have standardized treatment. Tsuji et al. sought to change this with a single-arm clinical trial to evaluate a regimen of GCs, tacrolimus, and CYC in a cohort of patients with MDA5+. The study demonstrated that the 12-month survival rate of patients in the novel treatment group was 85% compared to 33% in the control group, who underwent the traditional stepwise treatment of GCs then slow addition of other agents as needed [86]. Oral dosing of CYC initially starts at 2 mg/kg/day [70]. Of note, CYC is rarely used due to its significant toxicity profile, including the risk of infection, gonadal dysfunction, hemorrhagic cystitis, and malignancy [71].

#### 6.3.8. Anti-Fibrotic Agents

Immunosuppression is the key treatment for patients with myositis-associated ILD, and this makes sense when you consider that patients will almost always have evidence of inflammation on HRCT. The NSIP pattern on HRCT is the most commonly seen; however, there are also other patterns, such as fibrotic NSIP and UIP. Nintedanib and pirfenidone are FDA-approved for the treatment of idiopathic pulmonary fibrosis. There have not been trials to date that investigate the efficacy of anti-fibrotic agents in myositis-associated ILD; however, there is some evidence that these medications have therapeutic benefit. The SENS-CIS trial had 576 participants with systemic sclerosis, ILD, and fibrosis randomly selected to receive either placebo or nintedanib, an anti-fibrotic agent [87]. The study found that the rate of FVC % decline over a period of one year was significantly lower in the nintedanib group than in the placebo group (−52.4 mL/year vs. −93.3 mL/year) [87]. As a result of this trial and a few others like it, nintedanib has been approved by the US Food and Drug Administration (FDA) and the European Medicines Agency (EMA) for slowing the decline in FVC in patients with ILD associated with systemic sclerosis [87]. Further studies are warranted with patient populations with myositis to specifically see if nintedanib is proven to reduce the progression myositis-associated ILD.

#### 6.3.9. Janus Kinase Inhibitors

Janus kinase inhibitor (JAKis) inhibit cytokine production depending on which JAK proteins are inhibited, including IL-6, IL-11, IL-12, IL-17, IL-22, IFN-α, β, and γ [88,89]. Several studies highlight the efficacy of JAK inhibitors in treating MDA5+ DM. Kurasawa et al. performed a retrospective review of tofacitinib (TOF) in five patients with MDA5+ DM-associated ILD who were refractory to first-line treatment [74]. They reported that 60% of patients treated with TOF had a good response and survived [74]. Another study treated a group of 18 patients who had MDA5+ DM-associated ILD and were treated with GC and TOF. These patients were compared to historical controls who were treated with standard treatment and evaluated for survival [90]. Patients who received TOF treatment had a higher survival rate at 6 months when compared to the historical controls [90]. The adverse effects associated with TOF are infections, typically viral infections such as herpes zoster and cytomegalovirus reactivation [71].

#### 6.3.10. CD-19-Targeted Chimeric Antigen Receptor T cells

CD-19-targeting-chimeric antigen receptor (CAR) T cells represent a novel therapy for B cell malignancies. However, recently, patients with SLE and ASyS have also been successfully treated with CAR T cells as therapy. Compared to rituximab, which targets B cells, CD-19-targeting CAR T cells may provide a therapeutic benefit by causing a synergistic effect on B cell depletion and the co-targeting of B cells and plasmablasts [91]. An adverse effect of this therapy regardless of what disease is being treated is cytokine release syndrome, and this can vary in severity [91]. However, a retrospective review of patients with B cell lymphoma with concurrent rheumatic autoimmune disease demonstrated that patients treated with CAR-T cell therapy have better biochemical control of their rheumatic disease [92]. The emergence of this therapy is promising, but more dedicated research needs to be conducted.

#### 6.3.11. Plasmapheresis

Plasmapheresis has been poorly studied as a treatment option, but the studies that do exist are specifically for MDA5+ DM. There are several case studies that demonstrate that polymyxin B-immobilized perfusion treatment along with plasma exchange led to successful outcomes for patients with MDA5+ DM RP-ILD [5]. However, a large European cohort study found that plasma exchange therapy was not associated with better outcomes in patients with MDA5+ DM ILD [93]. Patients receiving plasma exchange had more severe disease, which may have affected the outcomes. Therefore, the benefits of plasma exchange therapy are still unknown and should be evaluated in further studies.

## 7. Conclusions

Myositis a large umbrella term for various inflammatory disease processes, four of which were extensively covered in this review: PM, DM, ASyS, and MDA5+ DM. Although these disease processes all share the commonality of muscle involvement, they also have several other manifestations, which can complicate clinical picture. This was evident in all three of our case studies, where each patient presented with proximal muscle weakness and myositis of varying severity, but they also had an assortment of other symptoms as well. In the first case report, the 47-year-old female also presented with heliotrope rash and mechanic’s hands, features that we discussed in this review as commonly seen in DM. In the second case report, the 49-year-old female came in with systemic symptoms of fatigue and weight loss as well as Raynaud’s, features we learned are not essential in the diagnosis of PM but have been shown to be associated via various antibodies. In the third case report, our 68-year-old patient had severe skin disease and cardiac manifestations; in our discussion regarding MDA5+ DM, we learned that severe mucocutaneous skin findings differentiate this disease process from traditional DM, and there can be various cardiac manifestations. In all three cases, serologic testing and HRCT were performed to confirm the diagnosis. As we have described in this review, myositis is a disease process that affects various organs in the body and patients require a multidisciplinary team for optimal outcomes. Patients may initially present to a rheumatologist; however, they may also present to a pulmonologist for dyspnea, dermatologist for rash, cardiologist for arrhythmia, or even their primary care physician. Prompt recognition and treatment of IIM is necessary by the physician as myositis-associated ILD is a cause of significant morbidity and mortality in affected patients. All of our patients presented with dyspnea and the treating physician had the acumen to order HRCT, which allowed for prompt treatment. In our case studies, the patients were treated with high-dose GCs as well as several other steroid-sparing agents and all were stable in their disease processes. Although there is no well-defined treatment algorithm at this time for myositis-associated ILD, what is known is that the earlier the disease process is recognized and treated, the better the outcomes are for patients. Treatment for myositis-associated ILD warrants prospective and randomized controlled trials to compare the efficacy of the agents being used and a better understanding of the disease process to find new and better therapeutic targets.

## Figures and Tables

**Table 1:** Classification of idiopathic inflammatory myopathies by Bohan and Peter [3].

Classification of Myositis, 1975
Primary idiopathic polymyositis (PM)
Primary idiopathic dermatomyositis (DM)
PM/DM associated with malignancy
Childhood myositis: DM in >90%, vasculitis is common
PM/DM associated with an underlying connective tissue disease (overlap myositis)

IBM had not been described and would have been classified as PM. MSAs had not been discovered, so patients with ASyS would have been classified as PM or DM.

**Table 2:** Classification of adult idiopathic inflammatory myopathies by Dalakas [11] and Senécal [12].

Classification of Myositis, 2017	
Pure classic dermatomyositis	30–35%
Polymyositis phenotype Pure polymyositis Autoimmune necrotizing myopathy (IMNM)	15–20% ≤5% 10–15%
Sporadic inclusion body myositis	≤5%
Overlap myositis	50%

Overlap myositis in this classification system occurs if there are any of the following features: Raynaud’s phenomenon, arthritis, mechanic’s hands, ILD, trigeminal neuropathy, lower esophageal dysmotility, features of scleroderma, SLE, or Sjögren’s syndrome.

**Table 3:** Classification of adult idiopathic myopathies, including myositis specific antibodies.

Classification of Myositis Including Myositis-Specific Antibodies
Adult polymyositis (PM)
Immune-mediated necrotizing myopathy (IMNM) Anti-SRP+ Anti-HMGCR+
Adult dermatomyositis Amyopathic dermatomyositis (AMD): dermatopathic rash without clinical myositis
Antisynthetase syndrome (ASyS): can occur with PM or DM or in patients without myositis
Myositis associated with malignancy (DM more common than PM)
Myositis in overlap with other autoimmune diseases
Inclusion body myositis (IBM): may occur in overlap with Sjögren’s syndrome

**Table 4:** Myositis-specific and myositis-associated antibodies, adapted from Long et al., 2019 [1].

Antibody	Antigen	Prevalence in Myositis (%)	Common Manifestations
Myositis-specific antibodies (MSAs)			
Anti-aminoacyl tRNA synthetase antibodies (Jo1, PL-7, PL-12, EJ, OJ, Zo, YRS)	Cytoplasmic aminoacyl tRNA synthetase enzymes	Anti-Jo-1: 9–24 Other ASAb < 5	Myositis, arthritis, Raynaud’s phenomenon, mechanic’s hands, ILD
Anti-MDA5	Melanoma differentiation-associated gene 5	10–48 (East Asia), 0–13 (USA and Europe)	CADM, skin ulcerations, RP-ILD
Anti-Mi2	Nucleosome-remodeling deacetylase complex	11–59	Classic DM skin findings (heliotrope rash, Gottron’s sign, shawl sign), ILD
Anti-SRP	Cytoplasmic signal recognition particle	5–13	Decreased cutaneous involvement, severe necrotizing myopathy, no increased risk of ILD
Anti-T1F1-γ	Transcriptional intermediary factor 1-gamma	13–31	Malignancy
Anti-NXP2	Nuclear matrix protein 2	1–17	Malignancy Calcinosis in JDM
Myositis-associated antibodies (MAAs)			
Anti-Ro-52	52 kDa ribonuclear protein complex	9–46	ILD, arthritis, mechanic hands, overlaps with pSS, SLE, SSc
Anti-PM/Scl-75/100	Nuclear exome complex (70 kDa, 100 kDa subunits)	4–11	PM-SSc overlap: inflammatory joint disease, Raynaud’s, mechanic hands, ILD
Anti-Ku	70 kDa, 80 kDa Ku heterodimers	1–3	Arthralgia, Raynaud’s, mechanic hands, ILD; overlaps with SSc, SLE

Abbreviations: CADM—clinically amyopathic DM; pSS, pSS—primary Sjogren’s syndrome; RP-ILD—rapidly progressive interstitial lung disease; JDM—juvenile dermatomyositis; SLE—systemic lupus erythematosus; SSc—systemic sclerosis/scleroderma.

**Table 5:** Frequency of various ASAbs in patients with ASyS, prevalence of associated clinical features and other commonly seen manifestations, adapted from Chatterjee et al., 2013 [27].

Antibody	Antigen	Prevalence in patients with ASyS (Zhao, 2022) (%) [31]	Myositis (De Souza, 2023) (%) [32]	ILD (De Souza, 2023) (%) [32]	Arthritis (Huang, 2019) (%) [6]	Other Manifestations
Jo-1	Histidyl-tRNA synthetase	63	15–30	70–80	74	Arthritis, Raynaud’s, mechanic hands
PL-7	Threonyl-tRNA synthetase	15	5–10	84	50	Infrequent muscle involvement, severe arthritis
PL-12	Alanyl-tRNA synthetase	8	<5	90	42	Pulmonary hypertension, esophageal involvement
OJ	Isoleucyl-tRNA synthetase	-	1	8	44	ILD
EJ	Glycyl-tRNA synthetase	14	1	>90	53	DM
KS	Asparaginyl-tRNA synthetase	-	1	90	-	Fever, usual interstitial pneumonia
Zo	Phenylalanine0tRNA synthetase	-	<1	4–38	-	Rash, arthritis
YRS	Tyrosyl-tRNA synthetase	-	<1	60–100	-	NSIP, Raynaud’s, arthralgia

**Table 6:** Comparison of two proposed classification criteria for ASyS, adapted from Marco et al., 2020 [38,39,40].

Connors et al., 2010 [39]	Solomon et al., 2011 [40]
Antisynthetase antibody plus one or more of the following: Myositis by Bohan and Peter’s criteria ILD not explained by other causes Arthritis Unexplained, persistent fever Raynaud’s phenomenon Mechanic’s hands	Antisynthetase antibody plus two major criteria or one major and two minor criteria. Major criteria Myositis by Bohan and Peter’s criteria ILD not explained by other causes Minor criteria Arthritis Raynaud’s phenomenon Mechanic’s hands

**Table 7:** MSAs’ and MAAs’ association with ILD.

Autoantibodies	Frequency in IIMs (%)	Frequency of ILD (%)
MSAs		
Anti-Jo-1	15–30	70–80
Anti-PL-7	5–10	84
Anti-PL-12	<5	90
Anti-Mi-2	4–10	4–38
Anti-NXP-2	2–30	3
Anti-MDA5	7–14	60 (European) 90 (Asian)
Anti-TIF1-γ	20–30	<10
Anti-SRP	3–13	10–20
Anti-SAE	<10	<10
MAAs		
Anti-Ro52	10–40	7–50
Anti-U1RNP	6–10	7–50
Anti-PM-Scl	3–10	25–80
Anti-Ku	<2	27–80

**Table 8:** MSAs’ and MAAs’ association with ILD (2023 American College of Rheumatology guideline for the treatment of interstitial lung disease in people with systemic autoimmune rheumatic diseases; myositis).

Medication	Outcomes	References
Glucocorticoids	Response rate: 37.5% to 52%	Fujisawa et al., 2023 [71]
MMF	Response rate: 54.5%	Sharma et al., 2017 [72]
AZA	Response rate: 54%	Sharma et al., 2017 [72]
CNI	Response rate in patient who failed AZA, methotrexate and MMF: 94%	Sharma et al., 2017 [72]
Rituximab	10% improvement in FVC in 50% of patients studied, no significant improvement in FVC in remaining patients	Allenbach et al., 2015 [73]
JAKi	Response rate: 60%	Kuraswa et al., 2018 [74]
CYC	Response rate: 64%	Moreno-Torres et al., 2023 [75]

Recommendations are unpublished and are all conditional recommendations.

## Data Availability

Not applicable.
